# 2-(Furan-2-yl)-1,3-bis(furan-2-ylmeth­yl)-1*H*-benzimidazol-3-ium chloride monohydrate

**DOI:** 10.1107/S1600536812040135

**Published:** 2012-09-26

**Authors:** David K. Geiger, H. Cristina Geiger, Jared M. Deck

**Affiliations:** a Department of Chemistry, State University of New York-College at Geneseo, 1 College Circle, Geneseo, NY 14454, USA

## Abstract

The title hydrated salt, C_21_H_17_N_2_O_3_^+^·Cl^−^·H_2_O, exhibits disorder in one of the furan rings. The major and minor components have a refined occupancy ratio of 0.844 (19):0.156 (19). The structure displays intermolecular hydrogen bonding involving the water molecule and the chloride anion. Close intermolecular C—H⋯Cl and C—H⋯(furan ring) inter­actions complete the hydrogen bonding.

## Related literature

For examples of the synthesis of substituted benzimidazolium salts, see: Wang & Chang (2006 ▶); Hoesl *et al.* (2004 ▶); Rivas *et al.* (2001 ▶, 2002 ▶). For examples of structures of other tris­ubstituted benzimidazolium salts, see: Ennajih *et al.* (2009 ▶, 2010 ▶); Akkurt *et al.* (2008 ▶, 2004 ▶); Smith & Luss (1975 ▶). For the structure of 1,3-bis(furan-2-ylmethyl)-1*H*-benzo[*d*]imidazol-3-ium chloride, see: Akkurt *et al.* (2009 ▶). For other related literature, see: Costache *et al.* (2007 ▶); Elmali *et al.* (2005 ▶); Hayakawa *et al.* (1996 ▶); Horton *et al.* (2003 ▶); Nahlé *et al.* (2012 ▶); Welton (1999 ▶).
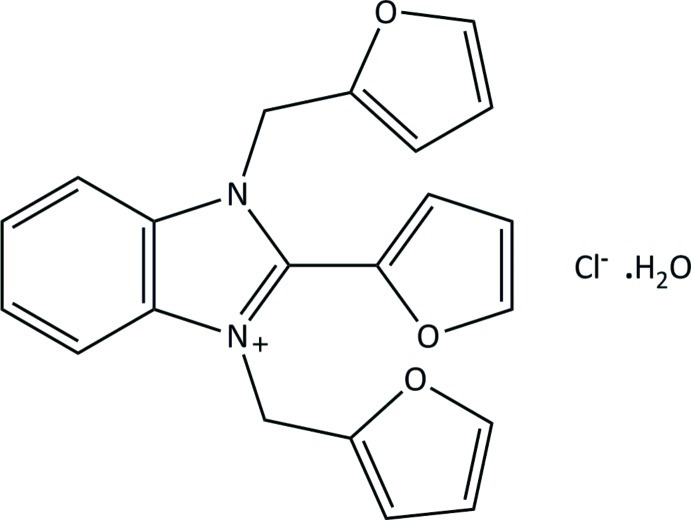


## Experimental

### 

#### Crystal data

C_21_H_17_N_2_O_3_^+^·Cl^−^·H_2_O
*M* *_r_* = 398.83
Triclinic, 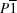

*a* = 9.4723 (12) Å
*b* = 9.9129 (14) Å
*c* = 11.2779 (16) Å
α = 97.980 (5)°
β = 110.359 (4)°
γ = 93.547 (4)°
*V* = 976.3 (2) Å^3^
*Z* = 2
Mo *K*α radiation
μ = 0.23 mm^−1^
*T* = 200 K
0.30 × 0.07 × 0.07 mm

#### Data collection

Bruker SMART X2S benchtop diffractometer
Absorption correction: multi-scan (*SADABS*; Bruker, 2009 ▶) *T*_min_ = 0.91, *T*_max_ = 0.98
9477 measured reflections
3419 independent reflections
2732 reflections with *I* > 2σ(*I*)
*R*_int_ = 0.036

#### Refinement

*R*[*F* ^2^ > 2σ(*F* ^2^)] = 0.045
*wR*(*F* ^2^) = 0.129
*S* = 1.05
3419 reflections
272 parameters
10 restraints
H atoms treated by a mixture of independent and constrained refinement
Δρ_max_ = 0.62 e Å^−3^
Δρ_min_ = −0.21 e Å^−3^

### 

Data collection: *APEX2* (Bruker, 2010 ▶); cell refinement: *SAINT* (Bruker, 2009 ▶); data reduction: *SAINT*; program(s) used to solve structure: *SHELXS97* (Sheldrick, 2008 ▶); program(s) used to refine structure: *SHELXL97* (Sheldrick, 2008 ▶); molecular graphics: *XSHELL* (Bruker, 2004 ▶) and *Mercury* (Macrae *et al.*, 2008 ▶); software used to prepare material for publication: *publCIF* (Westrip, 2010 ▶).

## Supplementary Material

Crystal structure: contains datablock(s) global, I. DOI: 10.1107/S1600536812040135/gg2097sup1.cif

Structure factors: contains datablock(s) I. DOI: 10.1107/S1600536812040135/gg2097Isup3.hkl

Supplementary material file. DOI: 10.1107/S1600536812040135/gg2097Isup4.mol

Supplementary material file. DOI: 10.1107/S1600536812040135/gg2097Isup4.cml

Additional supplementary materials:  crystallographic information; 3D view; checkCIF report

## Figures and Tables

**Table 1:** Hydrogen-bond geometry (Å, °)

*D*—H⋯*A*	*D*—H	H⋯*A*	*D*⋯*A*	*D*—H⋯*A*
O*W*—H*O*2⋯Cl1	0.85 (1)	2.35 (1)	3.189 (2)	174 (5)
O*W*—H*O*1⋯Cl1^i^	0.84 (1)	2.34 (1)	3.183 (2)	176 (4)
C9—H9⋯O3	0.95	2.49	3.278 (7)	141
C12—H12*A*⋯Cl1^ii^	0.99	2.77	3.694 (2)	155
C12—H12*B*⋯O1	0.99	2.39	3.098 (6)	127
C17—H17*B*⋯Cl1	0.99	2.61	3.594 (2)	176
C21—H21⋯O*W* ^iii^	0.95	2.29	3.168 (4)	153
C15—H15⋯O3^iv^	0.95	2.62	3.436 (3)	143
C15—H15⋯C18^iv^	0.95	2.85	3.788 (4)	170
C15—H15⋯C21^iv^	0.95	2.87	3.498 (4)	124

Symmetry codes: (i) 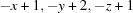; (ii) 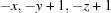; (iii) 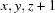; (iv) 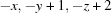.

**Fig. 1.**
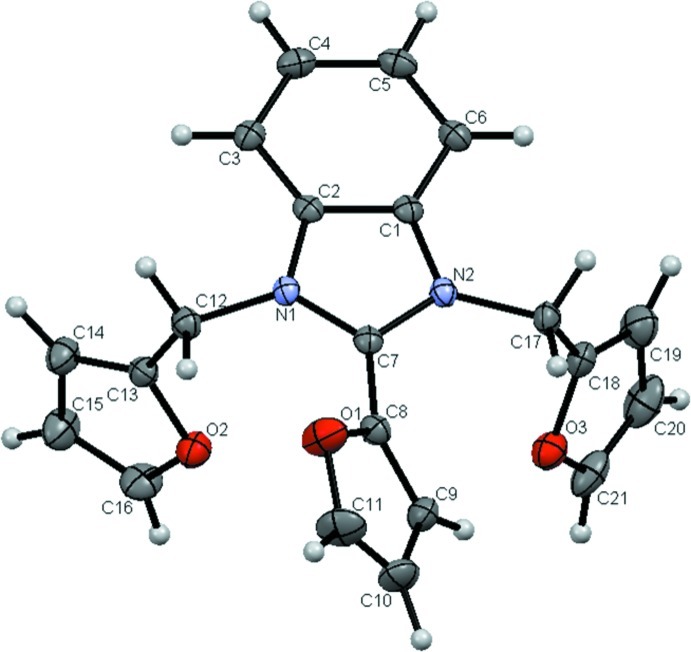
Perspective view of the benzimidazolium ion showing the atom labeling scheme. Displacement ellipsoids of non-hydrogen atoms are drawn at the 25% probability level. Only the major contributor to the disordered furan ring is shown.

**Fig. 2.**
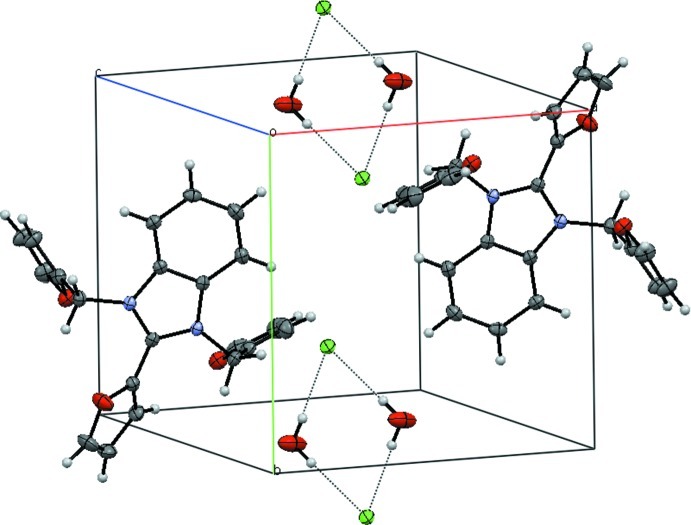
View of the unit cell showing the hydrogen bonding network. Only the major component of the disordered furan is shown. Ellipsoids are drawn at the 25% probability level.

**Fig. 3.**
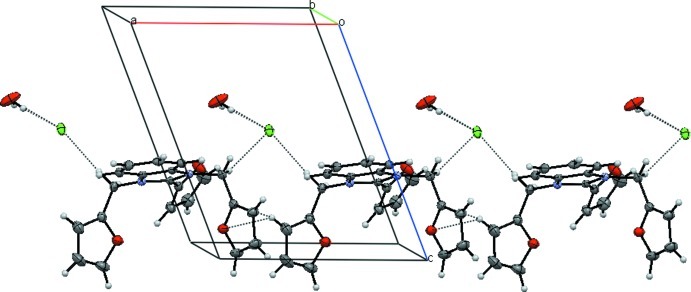
Close intermolecular C—H···Cl and C—H···O(furan) interactions resulting in a chain structure.

## supplementary crystallographic information

### Comment

Benzimidazolium salts have uses as room-temperature ionic liquids (Welton, 1999), surfactants (Costache *et al.*, 2007), inhibitors of corrosion (Nahlé *et al.*, 2012), anti-tumor (Elmali *et al.*, 2005) and other pharmacological agents (Horton *et al.*, 2003) and in nucleotide synthesis (Hayakawa *et al.*, 1996).

A perspective view of the benzimidazolium cation of the title compound is shown in Figure 1. The benzimidazole moiety is planar with the largest deviation being 0.011 (1) Å (N1). The furan rings are also planar with the largest deviations being 0.004 (3) Å (C11, major contributor to the disorder structure), 0.034 (13) Å (O1', minor contributor to the disordered structure), 0.011 (1) Å (C16), and 0.003 (2) Å(C20). The major component of the disordered 2-furan substituent is canted 45.1 (2)° from the mean plane of the benzimidazole ring system and the two components of the disordered furan form an angle of 22.0 (2)°. The two 2-furanylmethyl rings are on the same side of the benzimidazole with an angle of 82.79 (10)° between them.

Figure 2 shows the unit cell of the title compound. The hydrogen-bonding network involving the chloride anion and water are displayed. In addition to the O—H···Cl hydrogen bonding network, weak C—H···Cl interactions are observed. The H12A···Cl1 distance is 2.77 Å with C12···Cl1 = 3.627 (2) Å. Also, there is a weak C—H···aromatic interaction involving H15 and the furan ring containing O3. The closest approach is with O3 with H15···O3 = 2.63 Å. H15 is 2.602 (3) Å from the furan mean plane. These interactions result in chains of molecules parallel to the (0 0 1) plane as shown in Figure 3.

### Experimental

A single-crystal of the title compound was obtained during the attempted crystallization of 2-(2-furanyl)-1-(furanylmethyl)-1*H*-benzimidazole prepared *via* the aluminium trichloride catalyzed reaction of 1,2-diaminobenzene and furan-2-carbaldehyde in refluxing dichloromethane. The product was isolated by column chromatography on silica gel using an eluant that varied from 1:2 ethylacetate:hexanes to pure ethyl acetate.

The crystal used for the diffraction study was obtained by vapor diffusion of heptane into a methanol solution of the chromatographed product. Based on ^1^H NMR spectroscopy, the title compound makes up less than 5% of the bulk product.

### Refinement

All hydrogen atoms were observed in difference fourier maps. The H atoms were refined using a riding model with a C—H distance of 0.99 Å for the methylene carbon atoms and 0.95 Å for the phenyl and furan carbon atoms. All C—H hydrogen atom thermal parameters were set using the approximation *U*_iso_ = 1.2*U*_eq_.

The water oxygen atom was refined anisotropically. During the course of the refinement, *SHELXL* suggested that the oxygen atom of the water could be split into two atoms. However, attempts to refine the water assuming disorder result in no improvement in the GOF or the *R* values. The O—H distances were contrained to ~0.84 Å using *DFIX* and the H—O—H angles were restrained to ~104° using a DANG value of 1.34 Å between corresponding H atoms. The O—H hydrogen atom thermal parameters were set using the approximation *U*_iso_ = 1.2*U*_eq_.

During the later stages of refinement, elongated thermal ellipsoids were noted for one of the furan rings. A disorder model was developed in which the minor component of the furan ring was modeled using the metrics of the major components as a guide. The pivot atom (C8) was assumed to have full occupancy and so was not included in the disorder model. The minor four-atom components (O1', C9', C10' and C11') were constrained to planarity using FLAT. Corresponding bond distances of the minor component and major component were set equal using SADI and corresponding thermal parameters were held the same using EADP. All atoms were refined anisotropically with hydrogen atoms in calculated positions using a riding model. With these constraints, the site occupancy of the major component refined to 0.84 (2). Based on this model, the angle between the mean planes of the major and minor components of the disordered furan ring is 22.(2)°.

Improvement in the usual indicators occurred with the introduction of the disorder model. The *S* improved from 1.06 to 1.04. The *R* decreased from 0.046 to 0.045 and *wR* decreased from 0.132 to 0.129. However, an unusually large residual peak (0.61 e^-^/Å^3^) that is 1.00 Å from a hydrogen atom exists. The hydrogen atom in question (H12A) is involved in a close interaction with the chloride ion (2.78 Å). The next largest residual peak is 0.37 e^-^/Å^3^ and it is located 1.29 Å from H15, which is involved in a weak C—H···aromatic ring hydrogen bonding interaction (the closest approach is to O3 at 2.63 Å).

### Crystal data

**Table:**

C_21_H_17_N_2_O_3_^+^·Cl^−^·H_2_O	*Z* = 2
*M**_r_* = 398.83	*F*(000) = 416
Triclinic, *P*1	*D*_x_ = 1.357 Mg m^−^^3^
*a* = 9.4723 (12) Å	Mo *K*α radiation, λ = 0.71073 Å
*b* = 9.9129 (14) Å	Cell parameters from 3551 reflections
*c* = 11.2779 (16) Å	θ = 2.4–24.8°
α = 97.980 (5)°	µ = 0.23 mm^−^^1^
β = 110.359 (4)°	*T* = 200 K
γ = 93.547 (4)°	Needle, clear colourless
*V* = 976.3 (2) Å^3^	0.30 × 0.07 × 0.07 mm

### Data collection

**Table:**

Bruker SMART X2S benchtop diffractometer	3419 independent reflections
Radiation source: XOS X-beam microfocus source, Bruker SMART X2S benchtop	2732 reflections with *I* > 2σ(*I*)
Doubly curved silicon crystal monochromator	*R*_int_ = 0.036
Detector resolution: 8.3330 pixels mm^-1^	θ_max_ = 25.2°, θ_min_ = 2.0°
ω scans	*h* = −11→11
Absorption correction: multi-scan (*SADABS*; Bruker, 2009)	*k* = −11→11
*T*_min_ = 0.91, *T*_max_ = 0.98	*l* = −13→13
9477 measured reflections	

### Refinement

**Table:**

Refinement on *F*^2^	Primary atom site location: structure-invariant direct methods
Least-squares matrix: full	Secondary atom site location: difference Fourier map
*R*[*F*^2^ > 2σ(*F*^2^)] = 0.045	Hydrogen site location: inferred from neighbouring sites
*wR*(*F*^2^) = 0.129	H atoms treated by a mixture of independent and constrained refinement
*S* = 1.05	*w* = 1/[σ^2^(*F*_o_^2^) + (0.0633*P*)^2^ + 0.4042*P*] where *P* = (*F*_o_^2^ + 2*F*_c_^2^)/3
3419 reflections	(Δ/σ)_max_ < 0.001
272 parameters	Δρ_max_ = 0.62 e Å^−^^3^
10 restraints	Δρ_min_ = −0.21 e Å^−^^3^

### Special details

**Table:**

Geometry. All e.s.d.'s (except the e.s.d. in the dihedral angle between two l.s. planes) are estimated using the full covariance matrix. The cell e.s.d.'s are taken into account individually in the estimation of e.s.d.'s in distances, angles and torsion angles; correlations between e.s.d.'s in cell parameters are only used when they are defined by crystal symmetry. An approximate (isotropic) treatment of cell e.s.d.'s is used for estimating e.s.d.'s involving l.s. planes.
Refinement. Refinement of *F*^2^ against ALL reflections. The weighted *R*-factor *wR* and goodness of fit *S* are based on *F*^2^, conventional *R*-factors *R* are based on *F*, with *F* set to zero for negative *F*^2^. The threshold expression of *F*^2^ > σ(*F*^2^) is used only for calculating *R*-factors(gt) *etc*. and is not relevant to the choice of reflections for refinement. *R*-factors based on *F*^2^ are statistically about twice as large as those based on *F*, and *R*- factors based on ALL data will be even larger.

### Fractional atomic coordinates and isotropic or equivalent isotropic displacement parameters (Å^2^)

**Table:**

	*x*	*y*	*z*	*U*_iso_*/*U*_eq_	Occ. (<1)
Cl1	0.44526 (6)	0.74517 (6)	0.50520 (6)	0.0460 (2)	
OW	0.6147 (4)	0.9554 (2)	0.3947 (2)	0.0967 (9)	
HO1	0.597 (5)	1.033 (2)	0.423 (4)	0.145*	
HO2	0.575 (5)	0.902 (3)	0.429 (4)	0.145*	
C1	0.1566 (2)	0.5684 (2)	0.68521 (18)	0.0318 (5)	
C2	0.0112 (2)	0.5062 (2)	0.66052 (18)	0.0311 (5)	
C3	−0.0290 (3)	0.3649 (2)	0.62089 (19)	0.0365 (5)	
H3	−0.1283	0.3225	0.6042	0.044*	
C4	0.0851 (3)	0.2905 (2)	0.6075 (2)	0.0435 (6)	
H4	0.0638	0.1938	0.5817	0.052*	
C5	0.2319 (3)	0.3538 (3)	0.6310 (2)	0.0449 (6)	
H5	0.3062	0.2986	0.6197	0.054*	
C6	0.2706 (3)	0.4929 (2)	0.6698 (2)	0.0398 (5)	
H6	0.3695	0.5354	0.6853	0.048*	
N1	−0.07374 (18)	0.61074 (18)	0.68249 (15)	0.0303 (4)	
C7	0.0161 (2)	0.7312 (2)	0.72021 (18)	0.0312 (5)	
N2	0.15618 (19)	0.70877 (18)	0.72206 (15)	0.0316 (4)	
C8	−0.0297 (2)	0.8639 (2)	0.7526 (2)	0.0371 (5)	
C9	0.0340 (8)	0.9713 (6)	0.8502 (5)	0.0481 (14)	0.844 (19)
H9	0.1283	0.9784	0.9192	0.058*	0.844 (19)
C10	−0.0713 (7)	1.0725 (7)	0.8273 (7)	0.0587 (14)	0.844 (19)
H10	−0.0589	1.16	0.8787	0.07*	0.844 (19)
C11	−0.1853 (5)	1.0220 (6)	0.7243 (8)	0.0689 (16)	0.844 (19)
H11	−0.2715	1.0677	0.6884	0.083*	0.844 (19)
O1	−0.1656 (4)	0.8939 (6)	0.6734 (6)	0.0664 (14)	0.844 (19)
C9'	0.059 (5)	0.970 (3)	0.843 (3)	0.0481 (14)	0.156 (19)
H9'	0.1661	0.9845	0.887	0.058*	0.156 (19)
C10'	−0.057 (4)	1.053 (5)	0.850 (4)	0.0587 (14)	0.156 (19)
H10'	−0.0414	1.1381	0.9055	0.07*	0.156 (19)
C11'	−0.187 (3)	0.992 (3)	0.770 (3)	0.0689 (16)	0.156 (19)
H11'	−0.2783	1.0324	0.7541	0.083*	0.156 (19)
O1'	−0.1809 (18)	0.862 (2)	0.708 (3)	0.0664 (14)	0.156 (19)
C12	−0.2298 (2)	0.5859 (2)	0.6823 (2)	0.0389 (5)	
H12A	−0.29	0.5136	0.6089	0.047*	
H12B	−0.2793	0.6709	0.6721	0.047*	
C13	−0.2264 (2)	0.5425 (2)	0.8045 (2)	0.0378 (5)	
C14	−0.2923 (3)	0.4323 (3)	0.8311 (2)	0.0524 (7)	
H14	−0.3555	0.3564	0.7712	0.063*	
C15	−0.2481 (4)	0.4523 (3)	0.9676 (3)	0.0634 (8)	
H15	−0.278	0.393	1.0161	0.076*	
C16	−0.1564 (3)	0.5706 (3)	1.0140 (2)	0.0570 (7)	
H16	−0.1082	0.6076	1.1027	0.068*	
O2	−0.14159 (19)	0.63101 (18)	0.91660 (15)	0.0492 (4)	
C17	0.2888 (2)	0.8126 (2)	0.7527 (2)	0.0387 (5)	
H17A	0.2543	0.9046	0.7483	0.046*	
H17B	0.337	0.7931	0.6881	0.046*	
C18	0.4023 (3)	0.8125 (2)	0.8827 (2)	0.0424 (6)	
C19	0.5399 (3)	0.7716 (3)	0.9248 (3)	0.0663 (8)	
H19	0.5931	0.7321	0.8738	0.08*	
C20	0.5922 (4)	0.7991 (4)	1.0628 (3)	0.0774 (10)	
H20	0.6864	0.7807	1.1206	0.093*	
C21	0.4837 (4)	0.8549 (3)	1.0937 (3)	0.0652 (8)	
H21	0.4882	0.884	1.1789	0.078*	
O3	0.3639 (2)	0.86463 (18)	0.98518 (16)	0.0523 (5)	

### Atomic displacement parameters (Å^2^)

**Table:**

	*U*^11^	*U*^22^	*U*^33^	*U*^12^	*U*^13^	*U*^23^
Cl1	0.0399 (4)	0.0385 (3)	0.0571 (4)	0.0009 (2)	0.0175 (3)	0.0011 (3)
OW	0.197 (3)	0.0528 (13)	0.0828 (16)	0.0360 (17)	0.0950 (18)	0.0222 (12)
C1	0.0345 (12)	0.0383 (12)	0.0231 (10)	0.0040 (9)	0.0104 (9)	0.0074 (8)
C2	0.0344 (12)	0.0379 (12)	0.0215 (9)	0.0063 (9)	0.0091 (8)	0.0084 (8)
C3	0.0400 (13)	0.0381 (12)	0.0290 (11)	0.0011 (10)	0.0095 (9)	0.0075 (9)
C4	0.0601 (16)	0.0352 (12)	0.0345 (12)	0.0095 (11)	0.0155 (11)	0.0055 (10)
C5	0.0477 (15)	0.0508 (15)	0.0397 (13)	0.0198 (12)	0.0173 (11)	0.0086 (11)
C6	0.0360 (12)	0.0494 (14)	0.0363 (12)	0.0098 (11)	0.0145 (10)	0.0086 (10)
N1	0.0274 (9)	0.0353 (10)	0.0271 (9)	0.0025 (8)	0.0082 (7)	0.0067 (7)
C7	0.0326 (11)	0.0373 (12)	0.0233 (10)	0.0021 (9)	0.0091 (8)	0.0075 (8)
N2	0.0295 (10)	0.0366 (10)	0.0290 (9)	0.0019 (8)	0.0110 (7)	0.0067 (7)
C8	0.0356 (12)	0.0401 (13)	0.0367 (12)	0.0067 (10)	0.0136 (10)	0.0080 (10)
C9	0.041 (3)	0.0451 (15)	0.0502 (16)	−0.0033 (18)	0.0118 (16)	−0.0023 (12)
C10	0.075 (3)	0.033 (3)	0.071 (3)	0.0086 (18)	0.032 (2)	0.002 (2)
C11	0.072 (2)	0.055 (2)	0.078 (4)	0.037 (2)	0.020 (2)	0.012 (2)
O1	0.0532 (14)	0.059 (2)	0.067 (2)	0.0210 (14)	−0.0006 (13)	−0.0014 (16)
C9'	0.041 (3)	0.0451 (15)	0.0502 (16)	−0.0033 (18)	0.0118 (16)	−0.0023 (12)
C10'	0.075 (3)	0.033 (3)	0.071 (3)	0.0086 (18)	0.032 (2)	0.002 (2)
C11'	0.072 (2)	0.055 (2)	0.078 (4)	0.037 (2)	0.020 (2)	0.012 (2)
O1'	0.0532 (14)	0.059 (2)	0.067 (2)	0.0210 (14)	−0.0006 (13)	−0.0014 (16)
C12	0.0333 (12)	0.0452 (13)	0.0379 (12)	0.0045 (10)	0.0119 (10)	0.0084 (10)
C13	0.0338 (12)	0.0430 (13)	0.0342 (12)	0.0038 (10)	0.0101 (9)	0.0043 (10)
C14	0.0618 (17)	0.0512 (16)	0.0424 (14)	−0.0091 (13)	0.0191 (12)	0.0079 (12)
C15	0.083 (2)	0.0618 (18)	0.0477 (15)	−0.0101 (16)	0.0261 (15)	0.0175 (14)
C16	0.0727 (19)	0.0634 (18)	0.0328 (13)	0.0045 (15)	0.0153 (12)	0.0126 (12)
O2	0.0529 (11)	0.0516 (10)	0.0393 (9)	−0.0029 (8)	0.0134 (8)	0.0083 (8)
C17	0.0362 (13)	0.0388 (13)	0.0432 (12)	−0.0026 (10)	0.0176 (10)	0.0089 (10)
C18	0.0373 (13)	0.0388 (13)	0.0461 (13)	−0.0051 (10)	0.0116 (11)	0.0044 (10)
C19	0.0443 (16)	0.072 (2)	0.071 (2)	0.0068 (14)	0.0087 (14)	0.0062 (16)
C20	0.0531 (19)	0.074 (2)	0.075 (2)	−0.0040 (17)	−0.0146 (16)	0.0171 (17)
C21	0.070 (2)	0.0564 (18)	0.0466 (16)	−0.0170 (16)	−0.0038 (14)	0.0111 (13)
O3	0.0532 (11)	0.0541 (11)	0.0432 (10)	−0.0020 (9)	0.0109 (8)	0.0085 (8)

### Geometric parameters (Å, º)

**Table:**

OW—HO1	0.844 (10)	C9'—C10'	1.432 (18)
OW—HO2	0.847 (10)	C9'—H9'	0.95
C1—C2	1.391 (3)	C10'—C11'	1.297 (19)
C1—N2	1.397 (3)	C10'—H10'	0.95
C1—C6	1.397 (3)	C11'—O1'	1.388 (17)
C2—C3	1.395 (3)	C11'—H11'	0.95
C2—N1	1.398 (3)	C12—C13	1.489 (3)
C3—C4	1.384 (3)	C12—H12A	0.99
C3—H3	0.95	C12—H12B	0.99
C4—C5	1.410 (4)	C13—C14	1.345 (3)
C4—H4	0.95	C13—O2	1.383 (3)
C5—C6	1.373 (3)	C14—C15	1.429 (4)
C5—H5	0.95	C14—H14	0.95
C6—H6	0.95	C15—C16	1.338 (4)
N1—C7	1.347 (3)	C15—H15	0.95
N1—C12	1.483 (3)	C16—O2	1.365 (3)
C7—N2	1.352 (3)	C16—H16	0.95
C7—C8	1.449 (3)	C17—C18	1.486 (3)
N2—C17	1.482 (3)	C17—H17A	0.99
C8—O1'	1.341 (15)	C17—H17B	0.99
C8—C9	1.357 (4)	C18—C19	1.333 (4)
C8—C9'	1.358 (18)	C18—O3	1.373 (3)
C8—O1	1.366 (4)	C19—C20	1.439 (5)
C9—C10	1.444 (5)	C19—H19	0.95
C9—H9	0.95	C20—C21	1.322 (5)
C10—C11	1.290 (5)	C20—H20	0.95
C10—H10	0.95	C21—O3	1.369 (3)
C11—O1	1.371 (4)	C21—H21	0.95
C11—H11	0.95		
			
HO1—OW—HO2	103 (2)	C8—C9'—H9'	130.8
C2—C1—N2	106.81 (18)	C10'—C9'—H9'	130.8
C2—C1—C6	121.7 (2)	C11'—C10'—C9'	109 (4)
N2—C1—C6	131.4 (2)	C11'—C10'—H10'	125.5
C1—C2—C3	122.2 (2)	C9'—C10'—H10'	125.5
C1—C2—N1	106.73 (18)	C10'—C11'—O1'	114 (3)
C3—C2—N1	131.0 (2)	C10'—C11'—H11'	122.8
C4—C3—C2	115.7 (2)	O1'—C11'—H11'	122.8
C4—C3—H3	122.1	C8—O1'—C11'	97.6 (17)
C2—C3—H3	122.1	N1—C12—C13	110.44 (17)
C3—C4—C5	122.0 (2)	N1—C12—H12A	109.6
C3—C4—H4	119.0	C13—C12—H12A	109.6
C5—C4—H4	119.0	N1—C12—H12B	109.6
C6—C5—C4	122.0 (2)	C13—C12—H12B	109.6
C6—C5—H5	119.0	H12A—C12—H12B	108.1
C4—C5—H5	119.0	C14—C13—O2	110.3 (2)
C5—C6—C1	116.3 (2)	C14—C13—C12	133.2 (2)
C5—C6—H6	121.8	O2—C13—C12	116.5 (2)
C1—C6—H6	121.8	C13—C14—C15	106.2 (2)
C7—N1—C2	108.79 (17)	C13—C14—H14	126.9
C7—N1—C12	126.83 (18)	C15—C14—H14	126.9
C2—N1—C12	123.78 (18)	C16—C15—C14	106.9 (2)
N1—C7—N2	109.02 (18)	C16—C15—H15	126.6
N1—C7—C8	125.66 (19)	C14—C15—H15	126.6
N2—C7—C8	125.32 (19)	C15—C16—O2	110.8 (2)
C7—N2—C1	108.65 (17)	C15—C16—H16	124.6
C7—N2—C17	127.40 (18)	O2—C16—H16	124.6
C1—N2—C17	123.92 (18)	C16—O2—C13	105.8 (2)
O1'—C8—C9	109.7 (10)	N2—C17—C18	111.56 (18)
O1'—C8—C9'	120 (2)	N2—C17—H17A	109.3
C9—C8—O1	109.0 (4)	C18—C17—H17A	109.3
C9'—C8—O1	116 (2)	N2—C17—H17B	109.3
O1'—C8—C7	112.0 (9)	C18—C17—H17B	109.3
C9—C8—C7	133.6 (4)	H17A—C17—H17B	108.0
C9'—C8—C7	126 (2)	C19—C18—O3	109.8 (2)
O1—C8—C7	117.5 (2)	C19—C18—C17	133.6 (3)
C8—C9—C10	105.8 (5)	O3—C18—C17	116.7 (2)
C8—C9—H9	127.1	C18—C19—C20	106.6 (3)
C10—C9—H9	127.1	C18—C19—H19	126.7
C11—C10—C9	107.3 (6)	C20—C19—H19	126.7
C11—C10—H10	126.3	C21—C20—C19	106.7 (3)
C9—C10—H10	126.3	C21—C20—H20	126.6
C10—C11—O1	111.1 (5)	C19—C20—H20	126.6
C10—C11—H11	124.5	C20—C21—O3	110.3 (3)
O1—C11—H11	124.4	C20—C21—H21	124.9
C8—O1—C11	106.8 (3)	O3—C21—H21	124.9
C8—C9'—C10'	98 (3)	C21—O3—C18	106.7 (2)
			
N2—C1—C2—C3	179.89 (18)	C9—C10—C11—O1	−0.7 (6)
C6—C1—C2—C3	1.0 (3)	O1'—C8—O1—C11	96 (2)
N2—C1—C2—N1	0.3 (2)	C9—C8—O1—C11	−0.3 (4)
C6—C1—C2—N1	−178.61 (18)	C9'—C8—O1—C11	−11 (2)
C1—C2—C3—C4	−0.1 (3)	C7—C8—O1—C11	178.9 (3)
N1—C2—C3—C4	179.42 (19)	C10—C11—O1—C8	0.6 (5)
C2—C3—C4—C5	−0.7 (3)	O1'—C8—C9'—C10'	−4 (2)
C3—C4—C5—C6	0.7 (3)	C9—C8—C9'—C10'	−33 (9)
C4—C5—C6—C1	0.2 (3)	O1—C8—C9'—C10'	24 (2)
C2—C1—C6—C5	−1.0 (3)	C7—C8—C9'—C10'	−166.6 (15)
N2—C1—C6—C5	−179.6 (2)	C8—C9'—C10'—C11'	−1 (2)
C1—C2—N1—C7	−0.6 (2)	C9'—C10'—C11'—O1'	5 (3)
C3—C2—N1—C7	179.8 (2)	C9—C8—O1'—C11'	12.4 (16)
C1—C2—N1—C12	−172.23 (17)	C9'—C8—O1'—C11'	6 (2)
C3—C2—N1—C12	8.2 (3)	O1—C8—O1'—C11'	−80 (2)
C2—N1—C7—N2	0.7 (2)	C7—C8—O1'—C11'	171.4 (13)
C12—N1—C7—N2	172.00 (17)	C10'—C11'—O1'—C8	−7 (3)
C2—N1—C7—C8	−179.78 (18)	C7—N1—C12—C13	−92.9 (2)
C12—N1—C7—C8	−8.5 (3)	C2—N1—C12—C13	77.2 (2)
N1—C7—N2—C1	−0.5 (2)	N1—C12—C13—C14	−124.4 (3)
C8—C7—N2—C1	179.96 (18)	N1—C12—C13—O2	57.0 (3)
N1—C7—N2—C17	177.42 (17)	O2—C13—C14—C15	0.5 (3)
C8—C7—N2—C17	−2.1 (3)	C12—C13—C14—C15	−178.2 (3)
C2—C1—N2—C7	0.1 (2)	C13—C14—C15—C16	−1.4 (3)
C6—C1—N2—C7	178.9 (2)	C14—C15—C16—O2	1.8 (4)
C2—C1—N2—C17	−177.90 (17)	C15—C16—O2—C13	−1.5 (3)
C6—C1—N2—C17	0.9 (3)	C14—C13—O2—C16	0.6 (3)
N1—C7—C8—O1'	−17.5 (16)	C12—C13—O2—C16	179.5 (2)
N2—C7—C8—O1'	161.9 (15)	C7—N2—C17—C18	105.0 (2)
N1—C7—C8—C9	134.8 (4)	C1—N2—C17—C18	−77.4 (2)
N2—C7—C8—C9	−45.8 (5)	N2—C17—C18—C19	108.4 (3)
N1—C7—C8—C9'	147 (2)	N2—C17—C18—O3	−71.7 (3)
N2—C7—C8—C9'	−34 (2)	O3—C18—C19—C20	0.2 (3)
N1—C7—C8—O1	−44.2 (5)	C17—C18—C19—C20	−180.0 (3)
N2—C7—C8—O1	135.2 (5)	C18—C19—C20—C21	−0.4 (4)
O1'—C8—C9—C10	−26.4 (14)	C19—C20—C21—O3	0.4 (4)
C9'—C8—C9—C10	127 (10)	C20—C21—O3—C18	−0.3 (3)
O1—C8—C9—C10	−0.1 (4)	C19—C18—O3—C21	0.1 (3)
C7—C8—C9—C10	−179.1 (4)	C17—C18—O3—C21	−179.8 (2)
C8—C9—C10—C11	0.5 (5)		

### Hydrogen-bond geometry (Å, º)

**Table:**

*D*—H···*A*	*D*—H	H···*A*	*D*···*A*	*D*—H···*A*
O*W*—H*O*2···Cl1	0.85 (1)	2.35 (1)	3.189 (2)	174 (5)
O*W*—H*O*1···Cl1^i^	0.84 (1)	2.34 (1)	3.183 (2)	176 (4)
C9—H9···O3	0.95	2.49	3.278 (7)	141
C12—H12*A*···Cl1^ii^	0.99	2.77	3.694 (2)	155
C12—H12*B*···O1	0.99	2.39	3.098 (6)	127
C17—H17*B*···Cl1	0.99	2.61	3.594 (2)	176
C21—H21···O*W*^iii^	0.95	2.29	3.168 (4)	153
C15—H15···O3^iv^	0.95	2.62	3.436 (3)	143
C15—H15···C18^iv^	0.95	2.85	3.788 (4)	170
C15—H15···C21^iv^	0.95	2.87	3.498 (4)	124

Symmetry codes: (i) −*x*+1, −*y*+2, −*z*+1; (ii) −*x*, −*y*+1, −*z*+1; (iii) *x*, *y*, *z*+1; (iv) −*x*, −*y*+1, −*z*+2.
